# Glucagon-Like Peptide-1 (GLP-1)-Based Therapies and Hematological Malignancies: A Narrative Review of Current and Emerging Evidence

**DOI:** 10.7759/cureus.114008

**Published:** 2026-08-05

**Authors:** Hari Krishnan Nair

**Affiliations:** 1 Hematology and Medical Oncology, Western Michigan University Homer Stryker M.D. School of Medicine, Kalamazoo, USA

**Keywords:** glp-1 receptor agonist, hematological malignancy, immunomodulation, leukemia, lymphoma, multiple myeloma, myelodysplastic syndromes, obesity, type 2 diabetes

## Abstract

Initially introduced for the treatment of type 2 diabetes mellitus (T2DM), glucagon-like peptide-1 (GLP-1) receptor agonists and dual glucose-dependent insulinotropic polypeptide (GIP)/GLP-1 receptor agonists, have become integral to the management of both diabetes and obesity, with their therapeutic indications now extending to cardiovascular and renal diseases. As prescribing has surged, so too has interest in the oncologic implications of these agents. While the relationship between GLP-1-based therapies and solid tumors has been increasingly studied, their association with hematological malignancies, including leukemia, lymphoma, myelodysplastic syndromes (MDS), myeloproliferative neoplasms (MPN), and plasma cell dyscrasias, has only recently attracted dedicated investigation. This narrative review synthesizes the current preclinical, epidemiological, and clinical evidence linking GLP-1-based therapies to hematological malignancy risk, incidence, and outcomes. Emerging data from large retrospective cohort studies, findings from randomized controlled trial (RCT) network meta-analyses and observational pharmacoepidemiologic studies conducted in real-world settings suggest a predominantly protective association, though agent-specific heterogeneity exists. Putative mechanisms, including immunomodulation, inhibition of nuclear factor kappa B (NF-κB)-mediated inflammatory signaling, modulation of the bone marrow microenvironment, and reduction of obesity-mediated hematopoietic dysregulation, are discussed. Limitations of the existing evidence base, safety considerations during active cancer therapy, and directions for future prospective investigation are also addressed.

## Introduction and background

Initially approved for the management of hyperglycemia in type 2 diabetes mellitus (T2DM), glucagon-like peptide-1 receptor agonists (GLP-1RAs), including exenatide, liraglutide, dulaglutide, semaglutide, and the dual glucose-dependent insulinotropic polypeptide (GIP)/GLP-1 receptor agonist tirzepatide, have evolved into widely prescribed therapies. Their indications now extend beyond glycemic control to include chronic weight management, cardiovascular risk reduction, chronic kidney disease, metabolic dysfunction-associated steatohepatitis (MASH), and obstructive sleep apnea [1]. With the rapid adoption of these therapies, understanding their long-term oncologic safety profile has become a clinical imperative.

Hematological malignancies, encompassing leukemias, lymphomas, myelodysplastic syndromes (MDS), myeloproliferative neoplasms (MPN), and plasma cell neoplasms such as multiple myeloma (MM), represent a biologically heterogeneous group of cancers with substantial morbidity and mortality. Globally, hematological malignancies accounted for an estimated 1,311,104 new diagnoses and 700,205 deaths in 2022 [2]. Both obesity and T2DM, the primary causes for receiving GLP-1-based therapies, are independently established risk factors for these cancers. Meta-analyses have demonstrated that obesity increases the risk of non-Hodgkin lymphoma (NHL), Hodgkin lymphoma (HL), MM, and leukemia, including acute myeloid leukemia (AML) and chronic myeloid leukemia (CML), in a dose-dependent fashion with increasing body mass index (BMI) [3-5]. Similarly, T2DM is associated with a modestly but significantly elevated risk of NHL (odds ratio (OR) 1.22), leukemia (OR 1.22), and MM (OR 1.22), and a population-based Canadian study of over three million individuals confirmed that diabetes confers a 10% higher risk of hematological malignancies overall (adjusted hazard ratio (HR) 1.10) and a 36% higher all-cause mortality among those who develop such cancers [6,7].

Given that GLP-1-based therapies address both obesity and hyperglycemia, two modifiable risk factors for hematological cancers, and possess independent anti-inflammatory and immunomodulatory properties, the question of whether these agents influence hematological malignancy risk and outcomes has become a rapidly evolving area of investigation.

The objective of this narrative review is to summarize the current evidence regarding the association between GLP-1-based therapies and hematological malignancies, including biological mechanisms, epidemiological evidence, clinical outcomes in patients with established disease, safety considerations, and future research directions.

## Review

Methods: literature search strategy

This article is a narrative review. PubMed/MEDLINE, Embase, Scopus, and the Cochrane Central Register of Controlled Trials were searched from database inception to July 20, 2026, using combinations of the terms "GLP-1 receptor agonist", "glucagon-like peptide-1", "incretin", "semaglutide", "liraglutide", "dulaglutide", "exenatide", "tirzepatide", "hematologic/haematological malignancy", "leukemia", "lymphoma", "myelodysplastic syndrome", "myeloproliferative neoplasm", "multiple myeloma", "monoclonal gammopathy", and "clonal hematopoiesis". The 2025 and 2026 American Society of Clinical Oncology (ASCO) and American Society of Hematology (ASH) abstract archives, ClinicalTrials.gov, and the reference lists of retrieved articles were hand-searched. Eligible sources were English-language preclinical studies, observational cohort studies, randomized controlled trials (RCTs), meta-analyses and network meta-analyses (NMAs), and conference abstracts reporting hematological malignancy incidence, progression, or outcomes in relation to GLP-1-based therapies. Articles were excluded if they addressed only solid tumors without hematological endpoints, were non-English, or were duplicate reports of the same dataset. Study selection was performed by the single author; no formal risk-of-bias instrument or quantitative synthesis was applied, and evidence is therefore summarized qualitatively and presented hierarchically (preclinical, RCT-derived, observational, and conference abstract data are reported separately and labelled as such).

GLP-1 receptor biology in hematopoietic and immune cells

The GLP-1 receptor belongs to the class B family of G protein-coupled receptors and is primarily expressed in pancreatic beta cells, the gastrointestinal tract, the central nervous system, and the cardiovascular system. In the hematopoietic compartment, GLP-1R expression has been documented in thymocytes, splenocytes, and peripheral lymphocytes [8]. Hadjiyanni et al. demonstrated that GLP-1R signaling modulates lymphocyte proliferation and maintains peripheral regulatory T cells (Tregs) in murine models; GLP-1R-knockout mice exhibited hyperproliferative peripheral lymphocytes and a significantly reduced percentage of peripheral Tregs [8]. Collectively, these findings support a role for GLP-1R signaling in preserving immune homeostasis.

In a comprehensive receptor autoradiography study of 419 human tumors and 209 normal tissues, Körner et al. found that GLP-1R was expressed in various endocrine tumors and brain tumors but was notably absent in carcinomas and lymphomas. No GLP-1R expression was identified in lymph nodes or spleen [9]. This absence of direct receptor expression in lymphoid malignancies suggests that any protective effects of GLP-1RAs on hematological cancers are likely mediated through indirect mechanisms, including systemic immunomodulation, metabolic reprogramming, and modification of the bone marrow microenvironment, rather than direct antitumor signaling on malignant hematopoietic cells.

More recently, Rusznak et al. demonstrated that GLP-1R deficiency in recipient mice leads to profoundly increased graft failure following major histocompatibility complex (MHC)-mismatched allogeneic hematopoietic stem cell transplantation (HSCT), driven by enhanced host lymphocyte-mediated graft rejection rather than graft-versus-host disease. Depletion of CD90+ recipient T cells rescued engraftment, further supporting a model in which GLP-1R signaling restrains host immune-mediated rejection [10]. These findings identify GLP-1R as a novel regulator of allogeneic hematopoietic stem cell engraftment and suggest potential therapeutic applications in the transplant setting.

Mechanisms of potential hematoprotection

Several interconnected mechanistic pathways have been proposed to explain the emerging associations between GLP-1-based therapy use and reduced hematological malignancy risk.

Immunomodulatory and Anti-inflammatory Mechanisms

Persistent low-grade inflammation is a defining feature of both obesity and T2DM and is increasingly recognized as a driver of hematopoietic dysregulation, clonal hematopoiesis, and malignant transformation [11]. GLP-1RAs exert potent anti-inflammatory effects through multiple signaling cascades. Through the activation of cyclic adenosine monophosphate (cAMP)/protein kinase A (PKA) and adenosine monophosphate-activated protein kinase (AMPK) signaling cascades, GLP-1R signaling suppresses nuclear factor kappa B (NF-κB) activity, thereby limiting the transcription of proinflammatory cytokines, including tumor necrosis factor-alpha (TNF-α), interleukin-1 beta (IL-1β), and interleukin-6 (IL-6) [12-15]. GLP-1RAs also inhibit the nucleotide-binding domain, leucine-rich-containing family, pyrin domain-containing-3 (NLRP3) inflammasome and c-Jun N-terminal kinase (JNK) signaling [12,15,16]. These cytokines, particularly IL-6 and TNF-α, are directly implicated in the pathogenesis of MDS, MPN, and MM through their roles in dysregulated hematopoiesis, bone marrow stromal activation, and plasma cell survival [17].

Modulation of the Tumor Microenvironment and Immune Surveillance

In addition to suppressing inflammatory cytokine production, GLP-1RAs augment the activity of cytotoxic T lymphocytes and natural killer (NK) cells while promoting a shift in macrophage polarization from the pro-tumorigenic M2 phenotype toward the anti-tumorigenic M1 phenotype, and suppress myeloid-derived suppressor cells (MDSCs) [18,19]. In the context of immune checkpoint inhibitor (ICI) therapy, GLP-1R signaling has been shown to improve CD8+ T cell metabolic fitness, enhance central memory formation, and reduce lipid-induced T cell exhaustion [19]. These immunomodulatory effects may be particularly relevant to hematological malignancies, where immune evasion and microenvironmental immunosuppression are central to disease pathogenesis.

Obesity, Bone Marrow Adiposity, and Metabolic Reprogramming

Obesity increases bone marrow adiposity, creating a pro-inflammatory microenvironment that upregulates clonal hematopoiesis and supports leukemia development [11]. Bone marrow adipocytes promote chemokine-mediated tumor-cell migration, supply fatty acids for energy metabolism, enhance resistance to apoptosis through the release of IL-6, IL-1β, and TNF-α, and facilitate bone demineralization [20]. In contrast, adiponectin, an adipokine with anti-tumor properties, is diminished in obesity as well as in individuals with monoclonal gammopathy of undetermined significance (MGUS) who subsequently develop MM, and has been shown to promote myeloma cell death [20]. By reducing adiposity and improving metabolic parameters, GLP-1RAs may indirectly attenuate these adipose-dependent oncogenic pathways.

The analysis of more than 440,000 UK Biobank participants by Kapur et al. identified obesity-related characteristics as being associated with higher plasma IL-17A levels, diminished GLP-1R expression, and an elevated risk of myeloid malignancies. Mechanistic studies in a mouse model of protein tyrosine phosphatase non-receptor type 11 (PTPN11)-mutant leukemia further demonstrated that dual inhibition with an anti-IL-17A antibody and a GLP-1RA counteracted metabolic inflammation-induced leukemogenesis by limiting M2-like tumor-associated macrophages, restoring antigen presentation, re-establishing T-cell responses, and reducing leukemic disease burden [21]. This study provides direct mechanistic evidence linking GLP-1R agonism to suppression of obesity-driven myeloid leukemogenesis.

**Figure 1:**
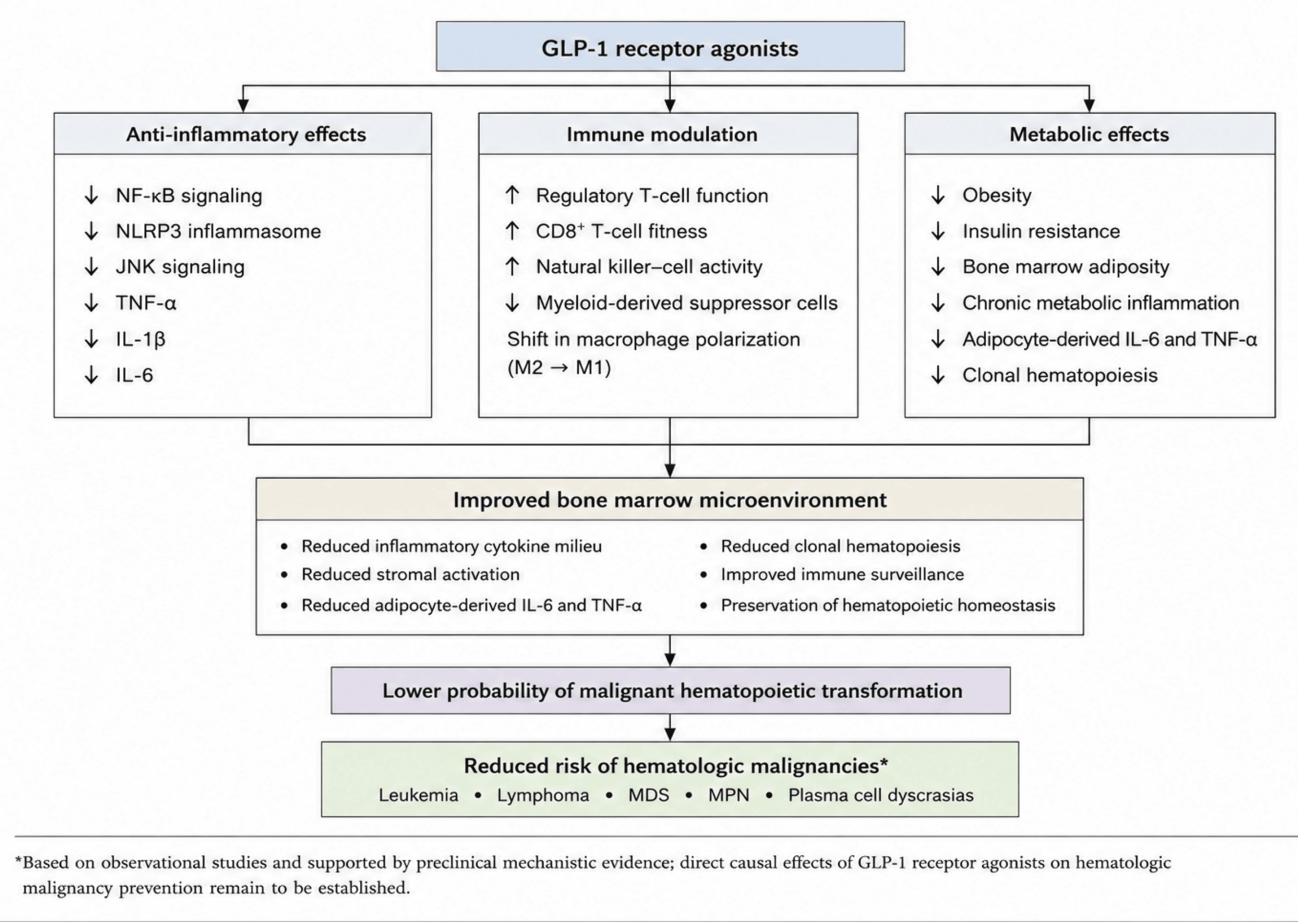
Proposed Mechanisms Linking GLP-1 Receptor Agonists to Reduced Risk of Hematologic Malignancies GLP-1, Glucagon-like peptide-1; CD8, cluster of differentiation 8; GLP-1RA, glucagon-like peptide-1 receptor agonist; IL, interleukin; JNK, c-Jun N-terminal kinase; M1, classically activated macrophage; M2, alternatively activated macrophage; MDS, myelodysplastic syndrome; MDSCs, myeloid-derived suppressor cells; MPN, myeloproliferative neoplasm; NF-κB, nuclear factor kappa B; NK, natural killer; NLRP3, nucleotide-binding oligomerization domain-, leucine-rich repeat-, and pyrin domain-containing protein 3; TNF-α, tumor necrosis factor alpha

Epidemiological evidence: GLP-1-based therapies and hematological malignancy risk

The evidence summarized below is predominantly observational. With the exception of post hoc analyses of RCTs pooled in the NMAs, none of the studies were designed to test causal effects on hematological malignancy incidence, and the reported associations should not be interpreted as establishing that GLP-1-based therapies prevent hematological cancers. Clinicians should not initiate or select these agents for chemopreventive indications on the basis of the present evidence.

Retrospective Cohort Studies

The first dedicated investigation of GLP-1-based therapies and hematological cancers was published by Ashruf et al. in *JAMA Network Open* in 2025. In this propensity score-matched cohort study, GLP-1RA use was compared with metformin and insulin in patients with T2DM. Compared with insulin, GLP-1RA use was associated with significantly lower risks of myeloid leukemia (HR 0.39; 95% confidence interval (CI) 0.25-0.60), lymphoid leukemia (HR 0.45; 95% CI 0.30-0.68), NHL (HR 0.42; 95% CI 0.30-0.58), MDS (HR 0.19; 95% CI 0.11-0.35), MPN (HR 0.50; 95% CI 0.41-0.61), monoclonal gammopathy (HR 0.68; 95% CI 0.52-0.88), MM (HR 0.49; 95% CI 0.31-0.76), and primary amyloidosis (HR 0.52; 95% CI 0.27-0.98). Compared with insulin therapy, GLP-1RA use was associated with a 54% reduction in the risk of developing hematological malignancies overall. Compared with metformin, which is considered to have potential cancer-protective effects itself, GLP-1RAs showed significantly reduced risks of MDS (HR 0.61; 95% CI 0.42-0.89) and MPN (HR 0.67; 95% CI 0.52-0.87), but no significant differences for leukemias, lymphomas, or plasma cell neoplasms [17].

Irons et al. conducted a large retrospective cohort study using the TriNetX database encompassing 405,454 individuals with T2DM, including 50,152 GLP-1RA users. GLP-1-based therapy was associated with a significantly lower risk of MM (HR 0.64, p=0.01), whereas no significant associations were observed for CML, AML, or MDS. Notably, this study also found that sodium-glucose cotransporter 2 inhibitor (SGLT2i) use was associated with significantly increased mortality in patients with MM (HR 2.27, p<0.001) and AML (HR 2.00, p=0.006), independent of heart or kidney failure, highlighting the importance of antidiabetic drug selection in patients with hematological malignancies [20].

A Medicare-based cohort study by Chen et al. presented at the 2026 ASCO Annual Meeting compared 14,606 GLP-1RA users with 14,606 propensity score-matched dipeptidyl peptidase-4 inhibitor (DPP-4i) users among older adults (mean age 77.3 years) with T2DM. GLP-1RA use was associated with reduced risks of MDS (HR 0.70; 95% CI 0.53-0.94), lymphoma (HR 0.71; 95% CI 0.57-0.89), and leukemia (HR 0.85; 95% CI 0.74-0.99). No statistically significant heterogeneity of treatment effect was detected across subgroups defined by age, sex, race/ethnicity, obesity status, or specific GLP-1RA agent [22].

Network Meta-Analyses of Randomized Controlled Trials

Two NMAs have specifically evaluated hematological malignancy risk across individual antidiabetic agents using data from RCTs. Lin et al. first published a pilot NMA of 55 RCTs (n=200,606) in *Biomolecules*, in which among individual GLP-1RAs, dulaglutide was associated with a significantly increased risk of hematological malignancies overall (OR 2.18; 95% CI, 1.14-4.19), whereas tirzepatide was associated with a significantly lower risk (OR 0.14; 95% CI, 0.03-0.60), particularly for lymphoma. No statistically significant associations were identified for SGLT2 inhibitors [23].

The same group subsequently published a larger, histopathology-stratified NMA in the *Journal of Hematology & Oncology*, analyzing 75 RCTs encompassing 270,471 participants. Dulaglutide again emerged as the only agent with a significantly elevated overall hematological malignancy risk (relative risk (RR) 2.17; 95% CI 1.14-4.17), while tirzepatide (RR 0.22; 95% CI 0.06-0.78) and the DPP-4 inhibitor linagliptin (RR 0.51; 95% CI 0.27-0.95) were linked to reduced overall risk. In histopathology-specific analyses, tirzepatide showed a significant protective association against NHL, whereas no agent demonstrated clear signals for leukemia or myeloma subtypes individually [24]. These findings underscore the importance of agent-specific and histology-stratified analyses rather than class-level generalizations.

Broader Cancer Risk Context

Ko et al. conducted a systematic review and meta-analysis of 48 randomized controlled trials encompassing 94,245 participants and reported that GLP-1RAs were unlikely to meaningfully alter the risk of most obesity-associated solid cancers, including thyroid, pancreatic, breast, and kidney malignancies (moderate-certainty evidence). A similarly minimal effect was observed for MM, although this conclusion was supported by low-certainty evidence [25]. In a separate systematic review and meta-analysis published in *Diabetes Research and Clinical Practice*, Ateiwi et al. analyzed 24 studies involving 3,960,974 patients and reported that GLP-1RA use was associated with a significantly lower overall risk of obesity-related cancers (RR 0.70; 95% CI, 0.54-0.89). The analysis also demonstrated a reduced risk of multiple myeloma (MM), along with several other obesity-related malignancies [26].

Plasma cell dyscrasias: MGUS and multiple myeloma

The relationship between GLP-1RAs and plasma cell disorders has attracted particular attention. MGUS is a premalignant plasma cell disorder with an estimated prevalence of approximately 3.2% among individuals aged 50 years or older and an estimated progression rate to MM of 0.5-1% per year [27,28]. Several studies have now examined whether GLP-1RA exposure modifies this trajectory.

Juranovic et al., in a TriNetX-based study presented at the 2025 ASCO Annual Meeting, found that among 5,901 MGUS patients with T2DM, GLP-1 agonist users had significantly lower MM rates at two, five, seven, and 10 years compared with non-users. Importantly, this protective effect was observed in patients with BMI ≥25 but not in those with normal BMI, suggesting that weight loss or related metabolic changes may mediate the protective effects [29].

Tentolouris et al. conducted a propensity score-matched study of 30,034 patients with MGUS and concurrent diabetes or overweight/obesity using the TriNetX network. GLP-1RA use was associated with significantly improved progression-free survival (HR 0.63; 95% CI 0.58-0.68), overall survival (HR 0.61; 95% CI 0.56-0.66), and a reduced risk of progression to symptomatic MM (HR 0.82; 95% CI 0.69-0.98) [30].

Chi et al. published a study in *JAMA Network Open* examining GLP-1RA use and major adverse cardiovascular and cerebrovascular events (MACCE) in patients with MGUS and diabetes. GLP-1RA use was associated with significant reductions in MACCE, driven primarily by decreases in all-cause mortality and new-onset heart failure. The authors noted that the intersection of obesity, diabetes, and chronic kidney disease in this population constitutes a well-established cardiovascular, renal, and metabolic axis in which GLP-1RAs have consistently demonstrated clinical benefit [27].

Ateiwi et al. presented data at the 2026 ASCO Annual Meeting comparing GLP-1-based therapies and plasma cell disorder risk across antidiabetic drug classes. GLP-1RA therapy was associated with significantly lower risk of both MGUS and MM compared with sulfonylureas (MGUS RR 0.59; MM RR 0.84) and SGLT2 inhibitors (MGUS RR 0.75; MM RR 0.62). In contrast, DPP-4i use was associated with increased MM risk compared with thiazolidinediones (RR 1.93) and SGLT2 inhibitors (RR 1.44) [31].

GLP-1-based therapies in patients with established hematological malignancies

Beyond cancer prevention, emerging evidence addresses the role of GLP-1RAs in patients with existing hematological malignancies. Using the TriNetX research network, Vemula et al. presented findings at the 2026 ASCO Annual Meeting examining the association between GLP-1RA therapy and clinical outcomes in adults with chronic lymphocytic leukemia (CLL) and T2DM. GLP-1RA use was associated with significantly lower risks of pneumonia (HR 0.53), sepsis (HR 0.51), thrombocytopenia (HR 0.63), as well as all-cause mortality (HR 0.30; 95% CI, 0.22-0.41) [32]. Cardiovascular mortality and intensive care unit admission rates were also significantly lower [32]. The authors noted that these outcomes were unlikely attributable to differential CLL therapy exposure, as treatment-free survival rates were high (80%-95%) across CLL therapy categories [32].

Albliwi et al. and Syal et al. independently examined GLP-1RA use in patients with MM and comorbid obesity or T2DM. Both studies found that GLP-1RA therapy was associated with significantly lower mortality (HR 0.44 and HR 0.42, respectively), reduced rates of sepsis, myocardial infarction, and ischemic stroke, and lower anemia rates. Patients on GLP-1RAs had higher hemoglobin levels, suggesting potential hematopoietic benefits [33,34].

Mahadevan et al. evaluated GLP-1RA effects on mortality and hospitalization in patients with T2DM and active cancer (including both solid and hematological malignancies) compared with metformin users. GLP-1RA recipients had significantly reduced mortality in both overall (HR 0.875) and new-start (HR 0.786) cohorts, along with lower rates of hospitalization, sepsis, major adverse cardiovascular events, and pneumonia [35].

Table 1 presents clinical studies that reported GLP-1-based therapies and hematological malignancies.

**Table 1:** Clinical Studies of GLP-1-Based Therapies and Hematological Malignancies AML, acute myeloid leukemia; BMI, body mass index; CI, confidence interval; CLL, chronic lymphocytic leukemia; CML, chronic myeloid leukemia; DPP-4i, dipeptidyl peptidase-4 inhibitor; EHR, electronic health record; GLP-1 RA, glucagon-like peptide-1 receptor agonist; HR, hazard ratio; MACCE, major adverse cardiovascular and cerebrovascular events; MDS, myelodysplastic syndrome; MGUS, monoclonal gammopathy of undetermined significance; MI, myocardial infarction; MM, multiple myeloma; MPN, myeloproliferative neoplasm; N, number of participants; NMA, network meta-analysis; OR, odds ratio; OS, overall survival; PFS, progression-free survival; RCT, randomized controlled trial; RR, relative risk; SGLT2i, sodium-glucose cotransporter 2 inhibitor; SR, systematic review; SU, sulfonylurea; T2DM, type 2 diabetes mellitus; TZD, thiazolidinedione; ↓, lower risk or event rate in exposed group; ↑, higher risk or event rate in exposed group.

Author (year)	Design/data source	N	Comparator	Outcome(s)	Key findings	Evidence type
Ashruf et al. (2025) [17]	Propensity-matched cohort, TriNetX	T2DM cohort	Insulin; metformin	Incident hematological malignancies	↓ Risk vs insulin across all subtypes (HR 0.19-0.68); vs metformin ↓ MDS and MPN only	Peer-reviewed
Irons et al. (2026) [20]	Retrospective cohort, TriNetX	405,454 (50,152 exposed)	Non-users; SGLT2i	Incidence, mortality	↓ MM (HR 0.64); null for CML/AML/MDS; SGLT2i ↑ mortality in MM and AML	Peer-reviewed
Chen et al. (2026) [22]	Propensity-matched cohort, Medicare	14,606 pairs	DPP-4i	MDS, lymphoma, leukemia	HR 0.70, 0.71, 0.85 respectively; no subgroup heterogeneity	Conference abstract
Lin et al. (2025) [23]	Pilot NMA of 55 RCTs	2,00,606	Placebo/active	Hematological malignancy	Dulaglutide OR 2.18; tirzepatide OR 0.14	Peer-reviewed
Lin et al. (2026) [24]	Histopathology-stratified NMA, 75 RCTs	2,70,471	Placebo/active	Subtype-specific risk	Dulaglutide RR 2.17; tirzepatide RR 0.22; linagliptin RR 0.51	Peer-reviewed
Ko et al. (2026) [25]	SR/meta-analysis, 48 RCTs	94,245	Placebo	Obesity-related cancers incl. MM	Little or no effect on MM (low certainty)	Peer-reviewed
Ateiwi et al. (2026) [26]	SR/meta-analysis, 24 studies	39,60,974	Various	Obesity-related cancers	RR 0.70 overall; ↓ MM	Peer-reviewed
Juranovic et al. (2025) [29]	Cohort, TriNetX	5,901 MGUS	Non-users	MGUS→MM progression	↓ MM at 2-10 y; effect confined to BMI≥25	Conference abstract
Tentolouris et al. (2026) [30]	Propensity-matched cohort, TriNetX	30,034 MGUS	Non-users	PFS, OS, progression	HR 0.63, 0.61, 0.82	Peer-reviewed
Chi et al. (2025) [27]	Cohort, TriNetX	MGUS + T2DM	Non-users	MACCE	↓ MACCE, driven by mortality and heart failure	Peer-reviewed
Ateiwi et al. (2026) [31]	Cohort, TriNetX	T2DM	SU, SGLT2i, TZD	MGUS, MM	↓ MGUS/MM vs SU and SGLT2i; DPP-4i ↑ MM	Conference abstract
Vemula et al. (2026) [32]	Cohort, TriNetX	CLL + T2DM	Non-users	Infection, mortality	↓ Pneumonia, sepsis, thrombocytopenia, mortality (HR 0.30)	Conference abstract
Albliwi et al. (2025) [33]; Syal et al. (2026) [34]	Propensity-matched cohorts, TriNetX	MM + T2DM/obesity	Non-users	Mortality, morbidity	Mortality HR 0.44 and 0.42; ↓ sepsis, MI, stroke, anemia	Conference abstracts
Mahadevan et al. (2026) [35]	Retrospective cohort	Active cancer + T2DM	Metformin	Mortality, hospitalization	HR 0.875 (overall), 0.786 (new-start)	Peer-reviewed

Agent-specific heterogeneity

A critical finding across multiple studies is the heterogeneity of hematological malignancy risk among individual GLP-1-based agents. The consistent signal of elevated hematological malignancy risk with dulaglutide in two independent NMAs (OR/RR ~2.17) contrasts sharply with the protective signal observed for tirzepatide (OR/RR 0.14-0.22) [23,24]. The mechanistic basis for this divergence remains unclear. Tirzepatide is a dual GIP/GLP-1 receptor agonist, and whether the additional GIP receptor agonism contributes to its protective profile, or whether differences in pharmacokinetics, receptor binding affinity, or downstream signaling bias account for these observations, requires further investigation [36]. Semaglutide and liraglutide have not shown statistically significant associations with hematological malignancy risk in the available NMA data, though a meta-analysis of semaglutide-specific RCTs and real-world studies reported a single case of B-cell lymphoma among real-world data with no overall increased cancer risk [37].

Safety considerations and clinical caveats

Several important caveats temper the interpretation of the current evidence. First, the overwhelming majority of data derive from retrospective observational studies and post hoc analyses of RCTs not designed to evaluate cancer endpoints. Residual confounding by indication, detection bias, and immortal time bias cannot be fully excluded despite propensity score matching [17,25]. Second, follow-up durations in most RCTs are insufficient to capture the latency period of hematological malignancies [25,38].

A practical concern for clinicians managing patients with hematological malignancies who are concurrently on GLP-1RAs is the risk of exacerbating cancer-associated cachexia and sarcopenia. Weight reduction achieved with GLP-1RAs is not limited to adipose tissue, with approximately 20%-30% of the total weight loss attributable to lean body mass [39,40]. In patients with cancer, diminished skeletal muscle mass has been linked to increased rates of severe treatment-related toxicity, more frequent dose modifications, and poorer survival outcomes [39]. Accordingly, Alati et al. recommended routine assessment of body composition together with dietetic intervention, resistance training, and ongoing monitoring when GLP-1RA therapy is initiated or maintained during active cancer treatment [39]. The American Diabetes Association (ADA) Standards of Care similarly advise against GLP-1RAs in patients experiencing unexplained weight loss or undernutrition and recommend monitoring for dehydration and excessive weight loss [41].

Additionally, the potential interaction between GLP-1RAs and SGLT2 inhibitors in the hematological malignancy setting deserves attention. While GLP-1RAs appear protective, SGLT2 inhibitor use has been associated with increased mortality in patients with MM and AML, potentially related to increased genitourinary infection risk in immunocompromised patients and worsening of kidney-associated complications [20].

Limitations

The evidence reviewed here is subject to several biases that are characteristic of pharmacoepidemiological research on antidiabetic agents and cancer, and these should temper interpretation of every effect estimate reported above.

Confounding by indication and channelling: GLP-1-based therapies are preferentially prescribed to patients with better renal function, fewer competing comorbidities, and longer anticipated survival, while insulin comparators identify patients with advanced, poorly controlled disease. Propensity score matching can balance only measured covariates, and residual confounding by frailty, performance status, cumulative glycemic burden, and adiposity trajectory is likely to persist.

Healthy-user and healthy-adherer effects: Patients who initiate and persist with injectable weight-management therapy differ systematically in health-seeking behaviour, cancer screening uptake, and socioeconomic status. The implausibly large mortality reductions reported in some cohorts (for example, HR 0.30 in CLL) are more consistent with this bias than with a drug effect [32].

Immortal time and prevalent user bias: Where exposure is defined after cohort entry without a corresponding landmark or time-varying treatment definition, follow-up during which the outcome cannot occur is misattributed to the exposed group, biasing estimates toward benefit. Not all included studies specified new-user designs or lag periods.

Detection and surveillance bias: GLP-1-based therapy users undergo more frequent laboratory monitoring and clinical contact, which could increase ascertainment of MGUS, cytopenias, and paraproteinemia in the exposed group, biasing incidence estimates upward, while also enabling earlier intervention that improves apparent outcomes.

Reverse causation: Undiagnosed hematological malignancy causes weight loss, anemia, and clinical deterioration, which both discourage GLP-1-based therapy initiation and prompt discontinuation. Without adequate exposure lag periods, this mechanism alone can generate apparently protective associations.

Competing risks and informative censoring: Comparator groups, particularly insulin and SGLT2 inhibitor users, have substantially higher cardiovascular and all-cause mortality, so fewer survive long enough to be diagnosed with a hematological malignancy. Cause-specific hazard models that do not account for competing mortality will overstate protection.

Exposure misclassification: Federated electronic health record (EHR) networks capture prescriptions or orders rather than dispensing and adherence, do not reliably distinguish agent, dose, formulation, or indication, and rarely quantify cumulative exposure, precluding dose-response assessment.

Inadequate latency and follow-up: Median follow-up in the source studies is generally well below the latency of myeloid and lymphoid neoplasia, and RCT follow-up averages approximately three years, which is insufficient for carcinogenic endpoints.

Evidence-level heterogeneity: A substantial proportion of the outcome data derive from conference abstracts that have not undergone full peer review, lack complete methodological reporting, and may not be published in final form.

Data-source overlap: The studies by Ashruf et al., Irons et al., Juranovic et al., Tentolouris et al., Chi et al., Ateiwi et al., Vemula et al., Albliwi et al., and Syal et al. were all conducted within the TriNetX federated network and draw on overlapping contributing healthcare organizations and, in all likelihood, overlapping patients [17,20,27,29-34]. These analyses therefore do not constitute independent replication; concordant findings across them may reflect shared coding practices, shared patients, and shared structural limitations of a single data ecosystem rather than reproducibility across distinct populations. The Medicare-based analysis by Chen et al. and the RCT-derived network meta-analyses provide the only substantially independent corroboration currently available [22].

Reporting heterogeneity: Effect measures are reported inconsistently across sources (hazard ratios, relative risks, and odds ratios, with variable availability of confidence intervals, P values, absolute event counts, and heterogeneity statistics), which precludes formal comparison across studies; effect estimates in this review are therefore reported as published, with the measure type specified in each case, and should not be pooled informally by the reader.

Finally, this is a single-author narrative review without duplicate screening or formal risk-of-bias assessment, and selection bias in the identification of eligible literature cannot be excluded.

Future directions

Research on GLP-1-based therapies in hematological oncology is at an inflection point, with emerging observational evidence now requiring prospective clinical and mechanistic validation. Several key priorities emerge for future investigation. First, prospective, adequately powered clinical trials with cancer-specific primary endpoints are needed to establish causality and quantify the magnitude of any protective effect [38,42]. Second, mechanistic studies should elucidate the molecular pathways through which specific GLP-1-based agents differentially influence hematopoietic stem cell biology, clonal hematopoiesis, and immune surveillance [21]. Third, the role of GLP-1-based therapies as potential chemopreventive agents in high-risk populations such as patients with MGUS and metabolic comorbidities warrants dedicated clinical trials [29,30]. Fourth, the interaction between GLP-1-based therapies and standard hematological cancer therapies, including ICIs, requires systematic evaluation [19]. Finally, the development of biomarker-driven approaches integrating GLP-1R expression profiling, metabolic phenotyping, and immune characterization may enable precision strategies for leveraging GLP-1-based therapies in hematological oncology [36,43].

Concretely, the following are needed: (1) prospective cohort studies in incident users with active-comparator, new-user designs, protocol-specified lag periods, and adjudicated hematological endpoints; (2) randomized trials, most feasibly as prespecified secondary or long-term extension analyses of ongoing cardiometabolic outcome trials, with harmonized malignancy adjudication and pooling across programmes to accrue adequate events; (3) follow-up of at least five to 10 years to span the latency of myeloid and lymphoid neoplasia, with registry linkage after trial completion; (4) dedicated randomized chemoprevention trials in high-risk populations, most plausibly MGUS or clonal cytopenia of undetermined significance with obesity, using biologically informative surrogate endpoints such as change in involved:uninvolved free light chain ratio, M-protein trajectory, or variant allele fraction of clonal hematopoiesis; (5) biomarker studies profiling GLP-1 receptor expression, circulating IL-6, IL-1β, TNF-α, IL-17A, adiponectin, and immune cell phenotypes to identify who benefits; and (6) mechanistic laboratory research using clonal hematopoiesis and murine leukemia and myeloma models to compare selective GLP-1 receptor agonism with dual GIP/GLP-1 co-agonism head-to-head and to dissociate drug-specific effects from those of weight loss.

## Conclusions

The currently available evidence, which is predominantly observational, retrospective, and in several instances limited to conference abstracts, suggests an association between the use of GLP-1-based therapies and a lower observed incidence of several hematological malignancies, including leukemias, lymphomas, MDS, MPN, and plasma cell disorders, relative to other antidiabetic therapies. Whether this association is causal remains unresolved, and it may be explained wholly or partly by confounding by indication, healthy-user effects, competing mortality, and inadequate latency. Plausible biological mechanisms, principally anti-inflammatory, immunomodulatory, and adiposity-related rather than direct antitumor effects, have been described, but these remain largely preclinical and do not by themselves establish clinical benefit. In patients with established hematological malignancies, observational data suggest better survival and fewer infectious and cardiovascular complications among users, although the magnitude of these estimates raises concern for residual bias, and the potential for lean mass loss and exacerbation of cancer-associated cachexia warrants nutritional and body composition monitoring during active treatment. At present, these findings do not support prescribing GLP-1-based therapies for the prevention or treatment of hematological malignancy; however, they support prospective, adequately powered studies with cancer-specific endpoints and sufficient follow-up.
